# Leucine Supplementation: A Novel Strategy for Modulating Lipid Metabolism and Energy Homeostasis

**DOI:** 10.3390/nu12051299

**Published:** 2020-05-02

**Authors:** Lingyu Zhang, Fengna Li, Qiuping Guo, Yehui Duan, Wenlong Wang, Yinzhao Zhong, Yuhuan Yang, Yulong Yin

**Affiliations:** 1 Hunan Provincial Key Laboratory of Animal Nutritional Physiology and Metabolic Process, Key Laboratory of Agro-ecological Processes in Subtropical Region, Institute of Subtropical Agriculture, Chinese Academy of Sciences, Changsha 410125, China; zly_012@163.com (L.Z.); gqp9210@163.com (Q.G.); duanyehui@isa.ac.cn (Y.D.); wangwenlong2012@yeah.net (W.W.); yinyulong@isa.ac.cn (Y.Y.); 2 College of Advanced Agricultural Sciences, University of Chinese Academy of Sciences, Beijing 100039, China; 3 Laboratory of Animal Nutrition and Human Health, School of Biology, Hunan Normal University, Changsha 410018, China; 4 Guangdong Provincial Key Laboratory of Animal Nutrition Regulation, South China Agricultural University, Guangzhou 510642, China; yinzhaoz@163.com; 5 College of Animal Science and Technology, Hunan Agricultural University, Changsha 410128, China; yangyu709842339@163.com

**Keywords:** leucine, lipid metabolism, energy axis, adipokine, microbiota

## Abstract

Lipid metabolism is an important and complex biochemical process involved in the storage of energy and maintenance of normal biological functions. Leucine, a branched amino acid, has anti-obesity effects on glucose tolerance, lipid metabolism, and insulin sensitivity. Leucine also modulates mitochondrial dysfunction, representing a new strategy to target aging, neurodegenerative disease, obesity, diabetes, and cardiovascular disease. Although various studies have been carried out, much uncertainty still exists and further studies are required to fully elucidate the relationship between leucine and lipid metabolism. This review offers an up-to-date report on leucine, as key roles in both lipid metabolism and energy homeostasis in vivo and in vitro by acceleration of fatty acid oxidation, lipolysis, activation of the adenosine 5′-monophosphate-activated protein kinase (AMPK)–silent information regulator of transcription 1 (SIRT1)–proliferator-activated receptor γ coactivator-1α (PGC-1α) pathway, synthesis, and/or secretion of adipokines and stability of the gut microbiota.

## 1. Introduction

In recent years, leucine (Leu) has garnered considerable attention due in part to its anabolic effects on muscles and the anti-obesity effects that result from its beneficial impact on glucose tolerance, lipid metabolism, and insulin sensitivity. Hence, research has gradually increased regarding Leu metabolism. Leu has been shown to influence lipid and energy metabolism in vivo and in vitro by accelerating fatty acid oxidation as well as cytokine synthesis and/or secretion, favoring a reduction in adiposity. It has been posited that increasing energy expenditure and removing toxic lipids by increasing the prevalence and activity of Leu may be a promising therapeutic strategy to treat obesity and its consequent conditions, such as insulin resistance, diabetes, and cardiovascular disease. This review aims to summarize recent developments in the biological function of Leu and its supplementation as a means to the control of metabolic diseases. This review also provides a reference for scientific use in animal husbandry and in the regulation of carcass quality by summarizing the mechanism of action behind Leu-regulated lipid and energy metabolism in mammals.

## 2. Leu Metabolism

Leu, chemically known as α-aminoisohexanoic acid, was first isolated from cheese by Proust in 1819 [1]. Later, Braconnot crystallized it from the acid hydrolysate of muscle and wool and named it Leu [1]. As an essential amino acid as well as a branched-chain amino acid (BCAA), Leu is widely found in animal protein and dairy products such as milk, eggs, pork, beef, and chicken, as well as in beans [2,3,4]. Some plants and fungi are also rich in Leu, including whole grains, vegetables, oats, wheat germ, garlic, and black fungi [5,6]. Leu has strong oxidative capabilities, and its main physiological functions include regulation of protein metabolism [7] and supply of oxidative energy [8]. This energy supply can be used in special physiological periods such as hunger, lactation, stress, and exercise [9], as well as in regulating immune functions [10] and in lipid metabolism [11].

Researchers began studying the role of BCAAs in protein synthesis as early as the 1970s, with a particular focus on Leu [12]. By the end of last century, the effects of Leu on protein synthesis were observed by supplementing diets with Leu or inducing production of Leu [13]. In addition to its effects on protein synthesis, Leu has a strong effect on the energy and lipid metabolism [14]. These effects have been investigated in detail since the beginning of the current century [15]. Strikingly, the mammalian target of rapamycin complex 1 (mTORC1) protein kinase, which is a well-known activator of the mTOR pathway, is essential to Leu’s regulation of lipid synthesis. Changes in the mTORC1 signaling pathway following Leu treatment have been detected at the molecular level to elucidate the mechanism of action of mTORC1 and its effects on lipid metabolism [16].

In summary, a plethora of human, animal, and cell experiments have shown that Leu can promote homeostasis and lipid metabolism through comprehensive mechanisms that will be discussed in detail in this review.

### 2.1. The Decomposing Process of Leu Oxidation

**Figure 1:**
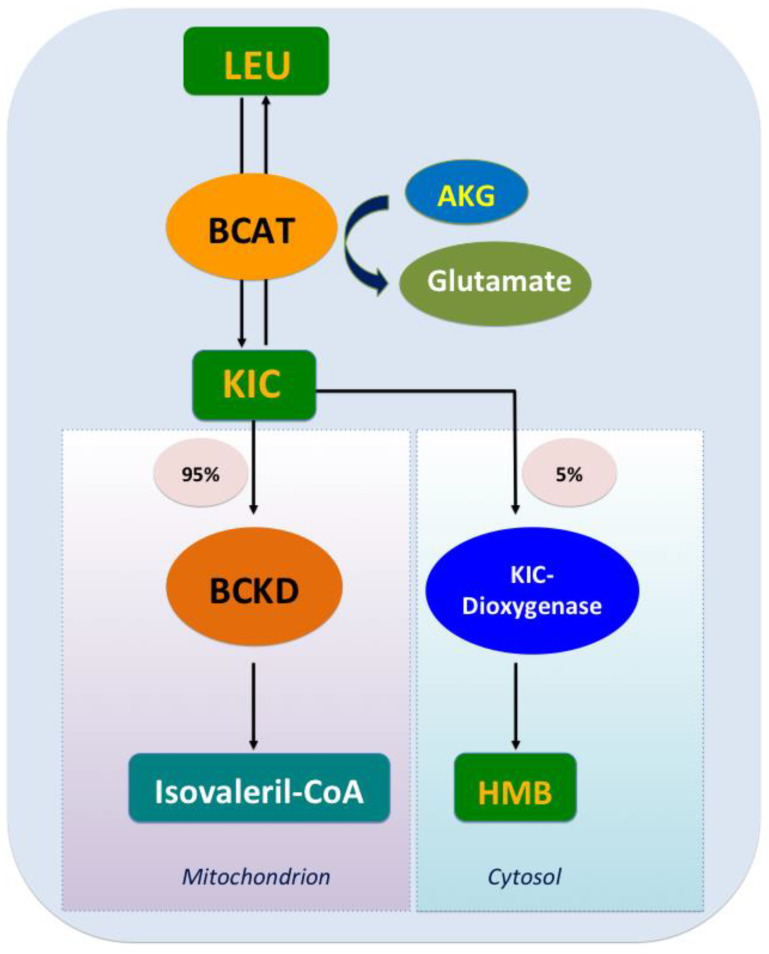
Leucine catabolism. After leucine ingestion, the reversible transamination of leucine to α-ketoisocaproate (KIC) occurs, through branched-chain amino acid (BCAA) transferase (BCAT) present in various tissues, and then approximately 5% of KIC is converted into β-hydroxy-β-methylbutyrate (HMB) by the cytosolic enzyme KIC dioxygenase, and about 95% ingested KIC is metabolized into isovaleryl-CoA, which is catalyzed predominantly by the mitochondrial branched chain a-keto acid dehydrogenase (BCKD).

The catabolism of Leu is a conserved regulator of physiological aging, participating in diverse physiological and pathological processes, including lipid metabolism [17]. The decomposition of Leu in mammals is complicated and involves two processes. Initially, the ingested Leu is catalyzed by BCAA transferase (BCAT) [18] to generate α-ketoisocaproate (KIC) and the precursor of β-hydroxy-β-methylbutyrate (HMB); this transamination is rapid and bidirectional (Leu+α-ketoglutarate<==>KIC+glutamate) [19]. Subsequently, KIC enters one of two metabolic pathways, generating either isovaleryl-CoA (90–95% of Leu metabolism) or HMB (5–10% of Leu metabolism). In the latter, KIC is irreversibly metabolized into HMB via KIC-dioxygenase; in the former, KIC undergoes irreversible and the rate-limiting oxidative decarboxylation through a series of reactions catalyzed by the branched chain a-keto acid dehydrogenase (BCKD) complex, which is regulated by BCKD kinase (BDK). Eventually, Leu is converted to acetoacetate acid and acetyl-CoA, which are intermediates of the tricarboxylic acid cycle (Figure 1). Excess KIC can be released into circulation and taken up by other organs such as the liver and the adipose tissue, where it is then resynthesized into BCAA or oxidized to generate adenosine triphosphate (ATP) [20].

### 2.2. Metabolites

Leu and its metabolites have been hypothesized to be regulatory signals for energy homeostasis. Studies indicate that Leu metabolites rather than Leu itself may be the signal for activation of mTOR [21]. In addition to Leu, HMB and KIC are direct activators of the silent information regulator of transcription 1 (SIRT1) enzyme [22]. It is evident that Leu has a pivotal role in partitioning energy from the adipose tissue to the skeletal muscle, resulting in a decreased energy storage in the adipocytes and increased fatty acid utilization in muscles. However, it remains unclear whether these effects are mediated by intact Leu or by KIC or by HMB. 

#### 2.2.1. KIC

Full consideration of the extant literature demonstrates that KIC is more efficacious than Leu at activating both the mTOR signaling and SIRT1 [23]. Both KIC and Leu suppress lipid anabolism in the adipocytes while promoting fatty acid oxidation (FAO). Moreover, treatment with KIC increases the oxidation of BCAA in cultured C2C12 myotubes. KIC increases complete FAO in skeletal muscle by inhibiting the BCKD kinase, resulting in strong activation of the BCKD complex and an increased flux through the BCAA oxidative pathway [24,25]. Increased free FAO decreases glucose utilization, and increased muscle mass can effectively enhance oxidation of fats. However, it is notable that KIC may be a double-edged sword, improving growth while at the same time increasing fatty acid synthesis by down-regulating the phosphorylation of adenosine 5′-monophosphate-activated protein kinase (AMPK), leading to negative effects on lipid metabolism in the adipose tissue [26]. As the mechanisms of KIC are complex and have not been fully explained, further study is imperative.

#### 2.2.2. HMB

Evidence has been provided, both in vitro and in vivo, that the endogenous conversion efficiency of Leu to HMB is approximately 5–10%. Despite this, the impact of HMB as a dietary supplement has been a focus of recent research on lipid metabolism. HMB is an interesting supplement in sports. In human trials, HMB intake in endurance training has an advantageous effect on the reduction of fat mass [27,28,29]. As athletes attempt to maintain a certain body mass, mostly through lowering the amount of adipose tissue, HMB supply may be a suitable choice for them to positively influence their physical performance. In one particularly interesting study, diet-induced obese mice were treated for 6 weeks with low (2 g/kg diet) or high (10 g/kg diet) doses of HMB, resulting in an increased adipose SIRT1 activity, uptake of muscle glucose and palmitate uptake, insulin sensitivity, as well as improvement in inflammatory stress biomarkers and reduced adiposity [30]. Bruckbauer et al. demonstrated that Leu /HMB in combination with low-dose resveratrol (200 nM) can synergistically activate SIRT signaling and stimulate energy metabolism by enhancing the FAO of adipocytes as well as insulin sensitivity. This resulted in increased activities of AMPK, SIRT1, and SIRT3 in murine muscle cells [31,32]. Furthermore, HMB has been reported to stimulate AMPK phosphorylation synergistically with metformin or resveratrol in C2C12 myotubes, resulting in a significant increase in insulin sensitivity and glucose tolerance in mice [31].

Dietary HMB supplementation may regulate the adipose tissue function, including FAO and lipolysis, with a concurrent increase of serum adiponectin concentration [33]. These effects may be partly mediated by the AMPKα–mTOR pathway and associated with mitochondrial biogenesis, the AMPK–SIRT1–proliferator-activated receptor γ coactivator-1α (PGC-1α) axis, and myokines [34] (discussed in detail in Section 5 and Section 6). Notably, HMB also has a critical role in regulating mitochondrial function, which relates to many diseases, such as aging, neurodegenerative diseases, obesity, diabetes, and cardiovascular disease [35]. Treatment of myotubes with a dose of HMB (50 mM) for 24 h significantly increased mitochondrial mass, respiration capacity, and biogenesis, and was superior to the effects observed with treatment of Leu (0.5 mM) [36]. HMB administration reversed the high-fat diet (HFD)-induced whitening and promoted the browning of brown adipose tissue (BAT), also demonstrating beneficial effects on the development of obesity and/or glucose homeostasis [37]. Moreover, HMB supplementation stimulates AMPK indirectly by elevating adiponectin levels, resulting in the suppression of mTOR signaling, which subsequently inhibits fatty acid synthesis and promotes lipolysis, decreasing the total weight of fat in a growing pig model [34]. Taken together, these results suggest that HMB may modulate mitochondrial biogenesis and FAO via the AMPKα–SIRT1–PGC-1α axis in the adipose tissue.

## 3. Leu and Lipid Metabolism in Adipose Tissue

### 3.1. Leu and Fatty Acid Oxidation in Adipose Tissue

Leu has also been reported to inhibit lipogenesis [38], to promote lipolysis and FAO [39], and to significantly increase leptin secretion in adipocytes via the mTOR signaling pathway [40], favoring a reduction of adiposity. Dietary Leu reduces hyperglycemia [41] and high cholesterol caused by HFD, decreases body fat and the rate of fat production, and increases insulin sensitivity [17]. In athletes, HMB supplementation for four weeks of resistance training significantly decreased cardiovascular risk factors, as low-density lipoprotein or total cholesterol and triglyceride [42]. Moreover, Nissen et al. also summarized that dietary HMB in humans results in a decrease in triglyceride and low-density lipoprotein cholesterol, thus affecting cardiovascular functions [43]. Long-term low-dose supplementation with Leu reduces the body fat and the rate of fat production, increasing insulin sensitivity [44]. Increasing Leu during adipocyte differentiation decreases the lipid droplet coating protein levels around lipid droplets, elevates the phosphorylation level of the hormone-sensitive lipase, and promotes fat decomposition [30]. Recently, some researchers have emphasized fact that Leu oxidation may be required for the activation of mTOR, the cytosolic serine/threonine protein kinase that appears to mediate FAO, and consequently Leu may regulate the metabolism of adipose tissue via KIC or HMB, acting as nutrient sensors [45]. The FAO energy purveyance of the adipose tissue is mainly used for the turnover of protein in the muscle tissue [46], which is one of the reasons why Leu decreases fat deposition and reduces body weight, but the exact mechanisms behind these phenomena need further study. 

### 3.2. Leu Promotes Browning

Leu may promote browning and mitochondrial biogenesis in white adipose tissue (WAT) via the SIRT1–AMPK–PGC-1α axis [47]. Adipose tissue plays an important role in regulating whole-body energy metabolism through energy storage in the white adipocytes and energy dissipation in the brown and the beige adipocytes. Indeed, the accumulation of excess WAT has deleterious consequences for metabolic health, whereas the activation of BAT helps to balance glucose levels and increase energy consumption, conferring beneficial effects on adiposity [48], insulin resistance, and hyperlipidemia [49]. Thus, in stimulating the development of the beige adipocytes in WAT, “browning” may reduce the adverse effects of WAT and may help to improve metabolic health. It is known that Leu is indispensable for brown adipocyte differentiation and regulates the mTOR signaling pathway in the adipocytes. Although the mTOR signaling pathway is required for the white adipocyte differentiation, it is only essential for the first stage of brown adipogenesis [50]. Recently, gut microbiota have been shown to modulate both the browning of WAT and the activity of BAT [51]; this activity can be modulated by Leu (discussed in detail in Section 7). Leu supplementation has been shown to induce a nearly fourfold increase in the mRNA expression of uncoupling protein 1 (*UCP-1*), a brown fat-specific gene, in WAT [52]. Moreover, the development of beige adipocytes in WAT occurs after Leu supplementation, thus reducing the adverse effects of WAT and enhancing the total capacity for FAO and improving metabolic health [53]. 

### 3.3. Leu Modulates Lipid Metabolism Via Mitochondria

Adipose tissue has evidently been perceived as a fuel reservoir that provides the skeletal muscle and other organs with non-esterified fatty acids when exogenous nutrients are insufficient [54]. In recent years, there has been great interest in the role of Leu in the metabolism of the adipose tissue [33]. It is important to emphasize that many in vitro and in vivo experiments have shown that Leu modulates lipid metabolism, mainly by inhibiting fat synthesis and decomposition, and increasing energy consumption [11,14,55]. There is growing evidence to suggest that the mitochondria may play a key role in modulating adipocyte lipid metabolism [56,57,58]. Specifically, mitochondria are necessary for the substrate oxidation and ATP generation that provides energy for cellular functions [59]. In addition, increased mitochondrial abundance induced by the nuclear factor erythroid 2-related factor overexpression in the adipose tissue increases adiponectin synthesis and has been shown to stimulate fatty acid combustion [60]. 

**Figure 2:**
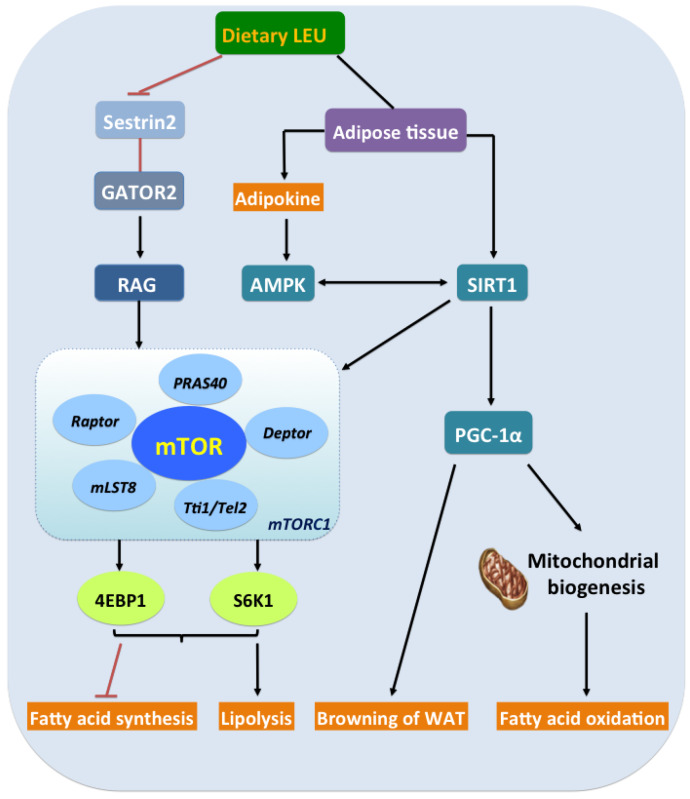
Dietary leucine effects on lipid metabolism in adipose tissues. After ingestion, leucine binds to Sestrin2, and then activates mammalian target of rapamycin complex 1 (mTORC1), facilitates lipolysis, and inhibits fatty acid synthesis by modulating the substrates such as translational inhibitor 4E-binding protein-1 (4EBP1) and S6 kinase 1 (S6K1). It also regulates adipokine synthesis/secretion and adenosine 5′-monophosphate-activated protein kinase (AMPK)–silent information regulator of transcription 1 (SIRT1)–proliferator-activated receptor γ coactivator-1α (PGC-1α) energy axis in adipose tissue, thus modulating mitochondrial biogenesis, promoting browning and fatty acid oxidation.

In the adipose tissue, the mTOR pathway appears to play an essential role in the differentiation of preadipocytes, adipose tissue morphogenesis, hypertrophic growth, and leptin secretion. Freshly isolated adipocytes contain a Leu-stimulated recognition site that is coupled to mTOR signaling and regulates lipid metabolism among other aspects of the mammalian physiology, including satiety [61], insulin secretion [62], and mitochondrial biogenesis [63]. This activity in large part is due to the activation of the mTORC1 protein kinase-a master growth controller, which is regulated by the Leu sensor Sestrin2 [64], a GATOR2-interacting protein that inhibits mTORC1 signaling. Indeed, Leu regulates the mTOR signaling pathway in adipocytes both in vitro and in vivo, and is more efficacious than other amino acids [65,66]. Moreover, mTOR balances energy metabolism by controlling the oxidative function of the mitochondria in an Akt-independent manner. In addition, BCAT activates the mTOR signaling pathway to promote mitochondrial biogenesis and ATP production, as well as to defend against oxidative stress by regulating the expression of related genes (Figure 2). That is to say, Leu promotes mitochondrial biogenesis, hence modulating lipid metabolism. 

Activating the serine/threonine protein kinase activity of mTOR leads to the phosphorylation of several substrates, including S6 kinase 1 (S6K1) and the translational inhibitor 4E-binding protein-1 (4EBP-1) [67]. In particular, translational inhibitor 4E-binding protein-1 (4EBP1) appears to be a novel regulator of adipogenesis and metabolism. Leu is capable of stimulating rapamycin-sensitive 4EBP1 phosphorylation in the adipocytes [68]. Briefly, Leu regulates the organization of adipocytes into tissue-like structures and the synthesis/secretion of leptin from adipose tissues through its regulation of mTOR signaling [69]. It has been shown that SIRT1 inhibition induces the acetylation of S6K1 and inhibits the mTOR-dependent phosphorylation of S6K1, suggesting an interaction between SIRT1 and mTOR [70] (Figure 2). To summarize, Leu plays an important role in stabilizing energy and metabolism through its regulation of SIRT1 and the mTOR signaling pathway, and thereby promotes mitochondrial biogenesis and modulates lipid metabolism.

## 4. Leu and Lipid Metabolism in Skeletal Muscle

The skeletal muscle is a principal site of usage of glucose and fatty acids, and is one of the primary tissues responsible for insulin resistance in obesity and type 2 diabetes [71]. The skeletal muscle plays a crucial role in energy homeostasis by clearing serum-free fatty acid, whole-body FAO, and lipid utilization [72]. There is a growing body of evidence suggesting that defective metabolism in muscle mitochondria and subsequent impaired ability to oxidize fatty acids may be a causative factor in the accumulation of intramuscular fat [59,73,74]. In particular, increased mitochondrial content and function is closely associated with enhanced oxidative capacity of muscle fibers, leading to an improved muscle health and overall health and well-being [75]. Conversely, mitochondrial dysfunction in the skeletal muscle is a major predisposing factor for obesity and its related metabolic disorders [76]. Thus, maintaining abundant and functional mitochondria in the skeletal muscle is of great importance for sustained health. Though cytosolic lipolysis and lipophagy are two sides of the same coin, lipid oversupply to skeletal muscle and consequent incomplete fatty acid oxidation has been irrefutably linked to skeletal muscle insulin resistance [77]. Furthermore, inhibition of fatty acid uptake into skeletal muscle mitochondria has been shown to attenuate insulin resistance in carnitine O-palmitoyltransferase (CPT)-1 knockout mice [78], although increasing lipid supply to skeletal muscle is not always beneficial and is highly dependent on the biological context. Leu mediates the adipocyte lipid metabolism to provide the skeletal muscle with an appropriate increased supply of free fatty acid, providing adequate energy substrates that support protein synthesis [79]. Therefore, Leu can increase oxygen consumption and counteract mitochondrial dysfunction, insulin resistance, and obesity. 

Fatty acids have been shown to increase the peroxisome proliferator-activated receptor γ coactivator-1α (PGC-1α) levels in muscle cells, which is associated with an increased mitochondrial metabolism [80]. SIRT1 and AMPK promote mitochondrial biogenesis and oxidative capacity, and prevent mitochondrial dysfunction in the skeletal muscle [81]. Notably, skeletal muscle FAO appears to be associated with mitochondrial biogenesis [82] and expression of certain genes, including those for PGC-1α and SIRT1 that regulate energy metabolism via modulation of thermogenesis, mitochondrial number, and FAO [80]. Leu modulates energy metabolism, in part, through the regulation of mitochondrial biogenesis, and promotes FAO and mitochondrial biogenesis [83]. Leu promotes partitioning energy from the adipocytes to the muscle cells, leading to decreased lipid storage in the adipocytes and increased fat utilization in the muscles [84]. Moreover, dietary Leu increases insulin sensitivity by promoting FAO in the skeletal muscle [85]. Zemel et al. indicated that Leu was able to directly activate SIRT1 by promoting the enzyme’s affinity for its substrates and nicotinamide adenine dinucleotide (NAD)^+^, resulting in an elevated mitochondrial biogenesis and FAO in both the adipocytes and the myotubes [79]. Liang et al. demonstrated that dietary Leu protected against HFD-induced mitochondrial impairment and stimulated mitochondrial biogenesis and energy partitioning from the adipocytes to muscle cells through SIRT1-mediated mechanisms [86]. Furthermore, feeding diet-induced obese mice with a low-dose of resveratrol in combination with Leu or HMB increased the adipose SIRT1 activity, muscle glucose, palmitate uptake, and insulin sensitivity; improved inflammatory stress biomarkers; and reduced adiposity [30]. In another study, dietary Leu intake via drinking water resulted in weight gain in mice fed an HFD, as well as a 25% decrease in the adiposity, decreasing diet-induced obesity, hyperglycemia, and hypercholesterolemia [87]. Donato et al. found that Leu supplementation decreased body fat along with demonstration of a proportional drop in leptin concentration [88]. Consistent with these observations, Leu has been found to markedly enhance the oxidative capacity and increase the mitochondrial density in the skeletal muscle, partially through the AMPK-mediated increases of PGC-1α via the SIRT1-dependent pathway [89]. Leu increases mitochondrial biogenesis and PGC-1α expression, suggesting that Leu may regulate the skeletal muscle energy metabolism, in part, by modulating the PGC-1α expression.

## 5. Leu and the Energy Axis, AMPK/SIRT1/PGC-1α

AMPK, a major sensor of cellular energy, can be activated by cellular stress to increase intracellular AMP [90]. AMPK is vital for energy metabolism, cell growth, and differentiation. When the activity of coenzyme A carboxylase is inhibited, activated AMPK can augment fatty acid oxidation, decrease glucose output, and affect cholesterol and total triglyceride synthesis, thus impairing mTOR signaling and subsequently inhibiting lipogenesis. In fact, by increasing the secretion and/or synthesis of leptin and adiponectin from adipocytes, Leu plays a distinct role in energy metabolism by indirectly activating AMPK [14]. Researchers have suggested that adding Leu to the diet of HFD-induced obese mice may increase the p-AMPK/AMPK ratio in the liver, preventing hepatic lipogenesis and lipid accumulation [91]. AMPK activity in adipocytes increased by 42% and 55% with the supplementation of resveratrol with either Leu or HMB, respectively, subsequently stimulating FAO and augmenting insulin sensitivity [30].

SIRT1 senses the energy or nutritional status of WAT through the NAD^+^/NADH ratio, and regulates WAT mobilization by regulating PGC-1α, mitochondrial biogenesis, glucose, and lipid homeostasis [92]. There is an interaction between SIRT1 and mTOR during lipid metabolism [93]. In SIRT1 knock-out mice (*SIRT1^−/−^*), the volume of adipocytes in the adipose tissue diminishes, decreasing the expression of adiponectin and leptin to 60% of the normal level and subsequently reducing the differentiation efficiency of the adipocytes [94]. Leu increases the activity of SIRT-1, whereas *SIRT-1* knock-down suppresses Leu-induced expression of the mitochondrial regulatory genes, indicating that Leu-induced mitochondrial biogenesis is partly mediated by SIRT1 [95]. It is important to note that Leu, as well as HMB and KIC, directly activate SIRT1, which lowers the activation energy for NAD^+^, increases FAO, reduces fat deposition, enhances insulin sensitivity, and consequently stimulates the metabolism [22]. Moreover, Leu regulates the *SIRT1* gene in humans, as well as the expression and activity of *SIRT1* in the adipocytes or the myocytes in obese people. In summary, SIRT1 possesses a pivotal function in the process of energy metabolism, and Leu is involved in the nutritional regulation.

PGC-1α is activated by the SIRT1-AMPK signals, and in turn targets the nuclear respiratory factors, which regulate the expression of a number of nuclear-encoded mitochondria proteins. Apart from mitochondrial biogenesis, PGC-1α plays a role in lipid metabolism through the regulation of the peroxisome proliferator-activated receptor γ (PPARγ). Phosphorylation mediated by AMPK and deacetylation by SIRT1 promote the expression of the peroxisome proliferator-activated PGC-1α, stimulating mitochondrial biosynthesis and metabolism, and increasing the FAO. Additionally, PPARγ and PGC-1α are also important for the differentiation and development of the brown and the beige adipocytes [96]. Activated mTOR signaling has been shown to promote mitochondrial biogenesis and oxidative metabolism by regulating PGC-1α [97]; thus BCAAs, including Leu, promote the expression level of PGC-1α [98].

**Figure 3:**
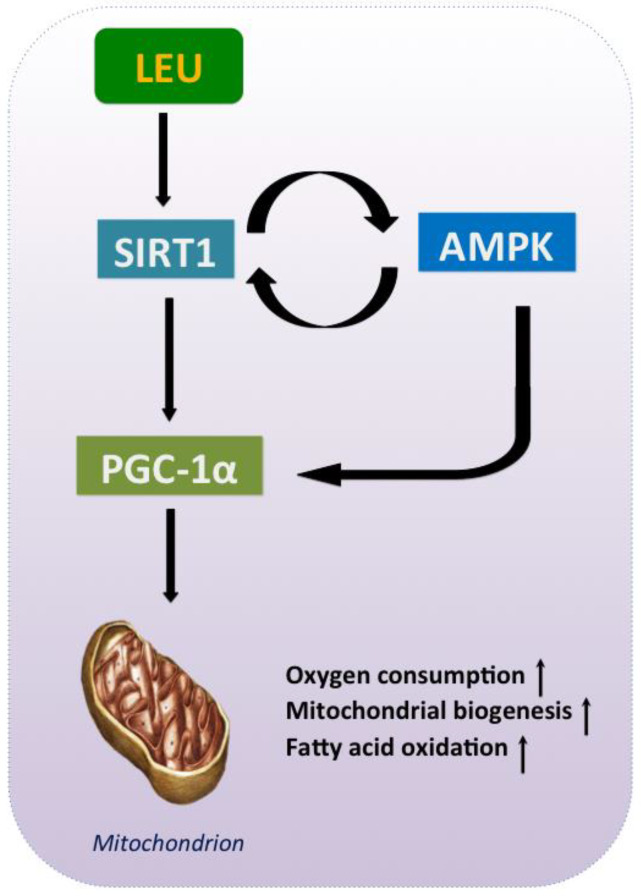
The SIRT1–AMPK–PGC-1α axis is involved in the leucine regulation in energy metabolism.

The SIRT1–AMPK–PGC-1α axis is a central signaling system that controls pathways involved in lipid metabolism; hence, it is a potential target for prevention and therapy of metabolic diseases. It has been well established that activation of the AMPK signaling pathway is related to the up-regulation of *SIRT1* and *PGC-1α* [99], with a bidirectional interaction between AMPK and SIRT1 [14]. AMPK activates SIRT1 by increasing the cellular NAD^+^ levels, and conversely, SIRT1 activation leads to the deacetylation of the protein kinase liver kinase B1 (LKB1), which promotes AMPK phosphorylation. The activation of SIRT1–AMPK signaling increases rates of fatty acid oxidation and represses lipogenesis, largely by modulating the activity of PGC-1α through deacetylation and phosphorylation, respectively [32]. Leu has a unique role as an activator of the AMPK–SIRT1–PGC-1α pathway, which confers its ability to modulate lipid metabolism and energy homeostasis (Figure 3). Leu, alongside HMB, regulates metabolism by stimulating the AMPK/SIRT1-dependent FAO [32]. First, Leu activates SIRT1, which results in the phosphorylation of AMPK, thus regulating FAO and mitochondrial biogenesis in the skeletal muscle [100]. As Banerjee et al. investigated, Leu and a low dose of metformin synergistically stimulate the AMPK–SIRT1 pathway by lowering the activation energy of NAD, inducing PGC-1α phosphorylation and activation, and thus up-regulating mitochondrial biogenesis and enhancing FAO [14]. In addition, there is evidence to suggest that Leu promotes browning and mitochondrial biogenesis in WAT via the SIRT1–AMPK–PGC-1α axis [101]. That is to say, Leu stimulates AMPK and SIRT1 and subsequently increases the expression and activity of PGC-1α, playing a central regulatory role in energy metabolism by upregulating oxidative metabolism and stimulating mitochondrial biogenesis.

## 6. Leu and Adipokines/Myokines

The adipose tissue and the skeletal muscle both produce cytokines, which may contribute to the cross-talk between the adipose and the muscle tissue that regulates energy partitioning. Apart from its roles in energy storage, the adipose tissue acts as a critical endocrine organ, producing a wide range of cytokines (adipokines) that are pivotal for the regulation of behavior and lipid metabolism [102]. Adipokines include several bioactive molecules released by WAT, such as leptin, adiponectin, angiotensin, tumor necrosis factor alpha (TNFα), and interleukin-6 (IL-6) [103]. Adipokines are particularly important for determining whether to oxidize glucose and fatty acids or not, thus contributing to lipolysis or the storage of triglycerides as fat [102].

Leptin is a gene-encoded adipokine associated with obesity that is synthesized nearly exclusively in the adipose tissue (both WAT and BAT) in mammals [104]. Leptin exerts a powerful influence on behavioral choice and body weight by regulating hunger and food consumption, as well as lipolysis, energy consumption, and body temperature. Moreover, leptin stimulates the oxidation of fatty acids and the uptake of glucose, and prevents the accumulation of lipids in the non-adipose tissues [40]. Leptin secretion in vitro is regulated at the mRNA translational level by mTOR and its agonist, Leu [105]. Thus, Leu can affect satiety by stimulating leptin secretion [106].

Circulating leptin is proportional to the amount of body fat [104], conversely the concentration of circulating adiponectin has an inverse relationship [107]. Adiponectin is primarily expressed in the adipose tissue and secreted from the adipose tissue [108], increasing fatty acid oxidation and reducing fat accumulation in several organs. It is a pleiotropic organ-protective protein that is exclusively produced by the adipocytes and is decreased in obesity. In addition, adiponectin enhances exosome biogenesis and secretion in obesity [109], and excess of exosomes is known to cause insulin resistance [110] and cardiovascular functions [111]. The way in which to stimulate this pathway for future therapeutics remains to be studied. Interestingly, the SIRT1–AMPK–PGC-1α axis has emerged as a regulator of adiponectin signaling and the subsequent lipid decreasing action of adiponectin [112]. Notably, Leu may increase adiponectin levels [84], thus indirectly stimulating AMPK [113], leading to the inhibition of the mTOR signaling in the adipose tissues [50], which may inhibit fatty acid synthesis or promote lipolysis, resulting in the reduction of fat mass [114].

It has been emphasized that leptin and adiponectin are the two most important fat-derived cytokines and have been known to modulate energy expenditure [115]. Indeed, leptin and adiponectin alter lipid partitioning by increasing fat oxidation and decreasing fatty acid incorporation into triacylglycerols. This implies that adipokines such as leptin and adiponectin play an important role in energy and lipid metabolism. Leu indirectly activates AMPK by increasing the secretion and/or synthesis of leptin or adiponectin from the adipocytes, which subsequently stimulates fatty-acid metabolism in muscles [113]. Moreover, HMB may lower the concentration of the circulating IL-6, thus stimulating the release of adipocyte fatty acids and muscle FAO [31]. Additionally, TNFα, which is expressed and produced by the adipocytes and increased in obesity, has been shown to induce muscle wasting [116]. 

Myokines have also received scientific attention due to their potential metabolic functions. Leu can regulate fat deposition in the adipose tissue via myokines highly expressed in the skeletal muscle, including IL-15 [34], the fibroblast growth factor [117], and irisin [118]. This increases lipolysis and fatty acid oxidation, and decreases fat deposition in the adipose tissue while increasing the growth of the skeletal muscle fiber, being mediated by the AMPK–mTOR pathway. Thus, reciprocal regulation between the adipose tissue and the skeletal muscle may exist, and Leu may control adiposity by regulating lipolysis in the adipose tissue and energy usage in the skeletal muscle.

## 7. Cross-Talk Between Leu and Intestinal Lipid Metabolism

### 7.1. Dietary Leu and Intestinal Metabolism

It is widely accepted that many aspects of metabolic health and metabolic homeostasis are affected by the gut. The gut barrier plays a major role in the prevention of infections, and is the first-line of defense against antigens derived from ingested food, bacteria, and viruses. Many bacterial species in the gut microbiome are capable of de novo manufacture of BCAAs [119]. The effects of Leu on the intestinal lipid and energy metabolism have begun to attract attention and focus [120]. Leu supplementation altered the energy metabolism and utilization of the porcine intestinal enterocytes by reducing glycolysis [121]. In the intestinal epithelial cells, Leu has been shown to activate several signaling pathways which are involved in the regulation of an organism’s metabolism. In particular, Leu supplementation was found to significantly regulate energy metabolism in the intestinal epithelial cells of a weaned piglet model by reducing the level of reactive oxygen species, whose homeostasis is vital to porcine health. This reduction in the reactive oxygen species is induced by switching from oxidative phosphorylation towards glycolysis through the mTOR–hypoxia-inducible factor-1alpha pathway [122].

Intestinal FAO is a key metabolic pathway that contributes not only to the energy homeostasis of the intestinal cells but also to the entire organism. The capacity of lipid catabolism in the small intestine was found to be higher in obesity-resistant mice [123], and the stimulation of the intestinal FAO after ingestion of Leu was associated with weight loss. Indeed, intestinal FAO is regulated by Leu. Specifically, Goichon et al. revealed that Leu supplementation may slow down FAO in the human duodenal mucosa, involving in four proteins related to lipid metabolism: HADHA (trifunctional enzyme subunit alpha), ACADVL (acylcoenzyme A dehydrogenase very long-chain), CPT2 (carnitine O-palmitoyltransferase), and FABP1 (fatty acid-binding protein liver) [124].

### 7.2. Leu and Microbiota

There has been a growing focus on the mechanisms by which diet, as dietary Leu, can modulate the composition and function of the gut microbiota, consequently regulating lipid metabolism and impacting health. The mammalian intestinal microbiota is composed of trillions of microbes that facilitate host health, including conferring colonization resistance against gastrointestinal disorders [125]. Moreover, gut microbes are active participants in the intestinal physiology, immunity, and energy metabolism [126]. As different microbiota are able to regulate plasma lipid levels [127], glucose homeostasis, and leptin sensitivity [128], the composition of the intestinal microbiome dramatically affects lipid metabolism. Clinical and preclinical studies indicate that dietary BCAAs, especially Leu, exert beneficial effects in mice and in humans, including, increasing mitochondrial respiration, reducing the production of oxygen radicals, influencing lipid metabolism, and eventually delaying age-dependent changes in the gut microbiota [129]. For example, Tiihonen et al. indicated that BCAAs were able to disrupt the homeostasis of the intestinal microbiota [130]. Reciprocally, the gut microbiota can modulate circulating BCAAs [131]. Interactions between dietary Leu, host, and microbiota are reciprocal, and dietary Leu has a critical influence on the composition of the gut microbial community; in turn, microbes influence the efficiency of harvesting energy from the ingested food.

Gut microbiota are highly associated with short-chain fatty acid (SCFA) production [132], as the main products of activity of the intestinal microbes are SCFAs, especially acetate, propionate, and butyrate [133], which are produced by the fermentation of ingested fibers. HMB exerts anti-obesity effects by shaping the composition of the gut microbiome, which tends to reverse the alteration of SCFAs and generates propionic acid, concomitantly improving microbial diversity and mediating lipid metabolism. The three branched-chain SCFAs (isobutyric acid, 2-methylbutyric acid, and isovaleric acid) are mainly derived from the catabolism of BCAAs [134]. These SCFAs have been well recognized to function as both a host energy source and as signaling molecules that link the metabolic activity of the gut microbiota to host energy homoeostasis, especially by regulating lipid metabolism [135]. As such, Leu may regulate the microbiota and intestinal lipid metabolism through the branched-chain SCFAs, and this phenomena must to be investigated further. SCFAs stimulate the expression of some cytokines, such as leptin, IL-10, and IL-18 [136]. Additionally, SCFAs regulate lipolysis in the adipocytes through G protein-coupled receptors, subsequently exerting further effects on lipid metabolism. HFD-induced obesity can be alleviated by regulating gut microbiota [137], as SCFAs were decreased in HFD-fed mice, alongside intestinal microbial dysbiosis and aggravative lipid accumulation. In contrast, an SCFA-enriched diet prevents and reverses HFD-induced metabolic abnormalities via a PPARγ-dependent switch from lipogenesis to fat oxidation [138]. 

The gut microbiota modifies amino acid metabolism to some extent, which has been hypothesized to affect many physiological functions. For example, Ridaura et al. demonstrated that the gut microbiota from obese subjects induced a notable increase in the circulating BCAAs, which are characteristic of an obese state [139]. It seems that the lipid metabolism induced by Leu is closely related to that by the intestinal microbiota [140], as gut microbes may assist in absorption of Leu in the intestinal epithelial cells and intervene in Leu metabolism [122]. Recently, fecal microbiota transplantation, which transplants the fecal microbiota from a donor into a recipient or into a germ-free mouse, has been an important area of research and a therapeutic approach for obesity [141]. This research method can also be applied to study lipid metabolism—transplantation of HMB-producing microbes has been observed to exert anti-obesity effects in a diet-dependent manner [142]. However, it remains unclear as to how Leu targets the gut microbiota to regulate lipid metabolism and prevent obesity, demonstrating that further studies will be needed to define the underlying mechanism.

## 8. Application in Livestock Production

With the continuous improvement in living standards, consumers continue to demand high-quality pork products. Increasing the lean rate of swine, reducing fat deposition, and thus improving feed utilization have always been goals of the swine industry. Excessive fat deposition negatively affects the quality of swine carcasses and consequently affects human health. Hence, nutritional regulation of lipid metabolism has become important in the field of animal husbandry. It is important to emphasize that Leu has a regulatory effect on the animal protein synthesis and lipid deposition. Numerous studies have demonstrated that Leu is a promising candidate for regulating lipid balance in the field of animal husbandry. For instance, Bai et al. indicated that low BCAA levels inhibited fatty acid synthesis and enhanced FAO in the liver of the female broiler chickens [143]. Moreover, Deng et al. demonstrated that optimum Leu levels improved flesh quality, partly by regulating antioxidant-related signaling molecules leading to an enhanced antioxidant capacity [144]. In growing pigs, Duan et al. showed that a low-protein diet with the appropriate ratios of BCAAs was able to increase growth, and as a result regulate lipid metabolism in the skeletal muscle [34]. Similarly, excess dietary Leu, KIC, and HMB may regulate energy homoeostasis, growth performance, and immune responses in weanling or growing pigs [145,146]. Therefore, clarification of the regulatory mechanisms of Leu on lipid metabolism in the adipose tissue may provide a new strategy for the nutritional regulation of amino acids, leading to the application of Leu supplementation as a means to improve livestock carcass.

## 9. Conclusions

To date, Leu, as well as its metabolites, have been shown to possess a promising role in lipid and energy metabolism by directly or indirectly increasing the oxidation of fatty acids and improving metabolic health. Leu supplementation results in beneficial effects in the adipose tissue, the skeletal muscle, and the intestine. Furthermore, Leu may have an additive effect that may alleviate mitochondrial dysfunction, representing a new therapeutic approach against aging, neurodegenerative diseases, obesity, diabetes, and cardiovascular disease. From the standpoint of the practitioner, Leu has potential to be used as a functional additive for both human health and production of livestock. However, it is necessary to carry out additional experiments to elucidate its regulatory mechanisms.

## Abbreviations

ACADVL	acylcoenzyme A dehydrogenase very long-chain
AMP	adenosine monophosphate
AMPK	(AMP)-activated protein kinase
ATP	adenosine triphosphate
BAT	brown adipose tissue
BCAA	branched chain amino acid
BCAT	BCAA transferase
CAS	Chinese Academy of Sciences
CoA	coenzyme A
CPT	carnitine O-palmitoyltransferase
BCKD	branched chain a-keto acid dehydrogenase
BDK	branched chain a-keto acid dehydrogenase kinase
FABP1	fatty acid-binding protein liver
FAO	fatty acid oxidation
GATOR2	GAP activity toward Rags 2
GLUT-4	glucose transporter-4
HADHA	tifunctional enzyme subunit alpha
HFD	high fat diet
HMB	β-hydroxy-β-methylbutyrate
IL	interleukin
ISA	Institute of Subtropical Agriculture
Leu	leucine
LKB1	liver kinase B1
KIC	α-ketoisocaproate
mRNA	messenger ribonucleicacid
mTOR	mammalian target of rapamycin
mTORC1	mammalian target of rapamycin complex 1
NAD	nicotinamide adenine dinucleotide
p-AMPK	phosphorylated AMPK
PGC-1α	peroxisome proliferator-activated receptor γ coactivator-1α
PPARγ	peroxisome proliferator-activated receptor γ
PPM1K	protein phosphatase 1K
SCFA	short-chain fatty acid
SIRT1	silent information regulator of transcription 1
STS	Science and Technology Service
S6K1	protein S6 kinase 1
TNFα	tumor necrosis factor alpha
UCP1	uncoupling protein 1
WAT	white adipose tissue
4EBP1	translational inhibitor 4E-binding protein-1
